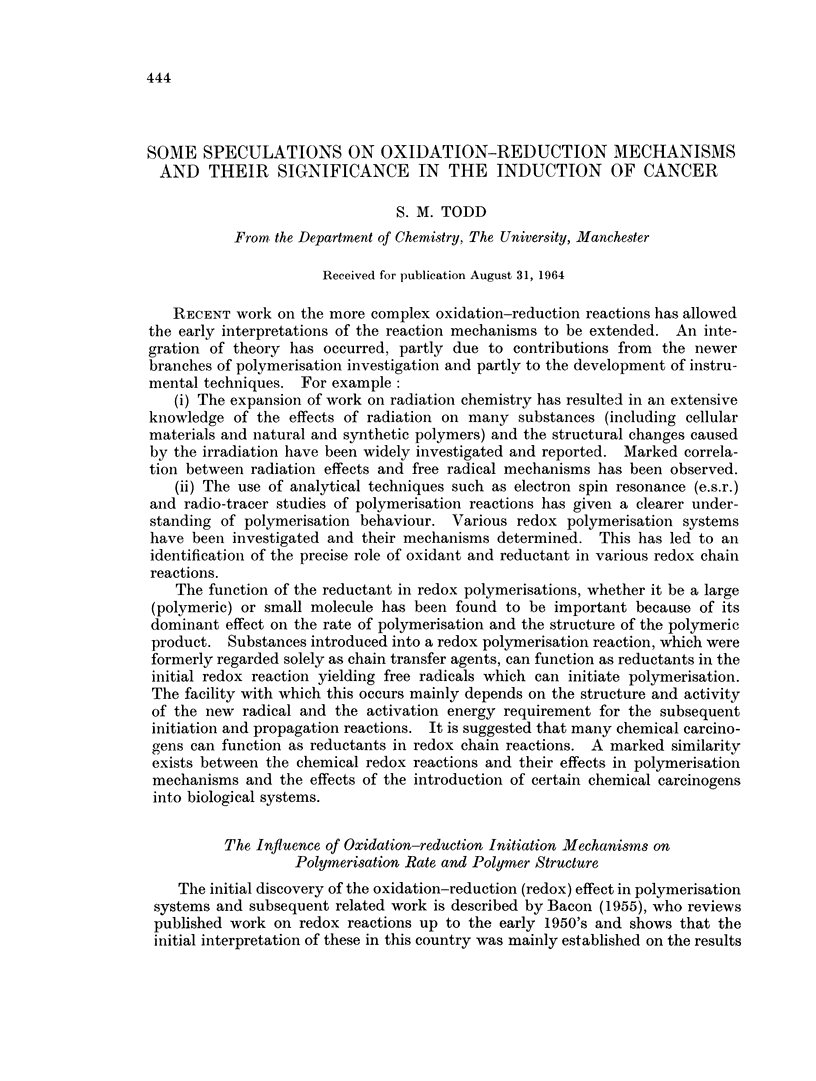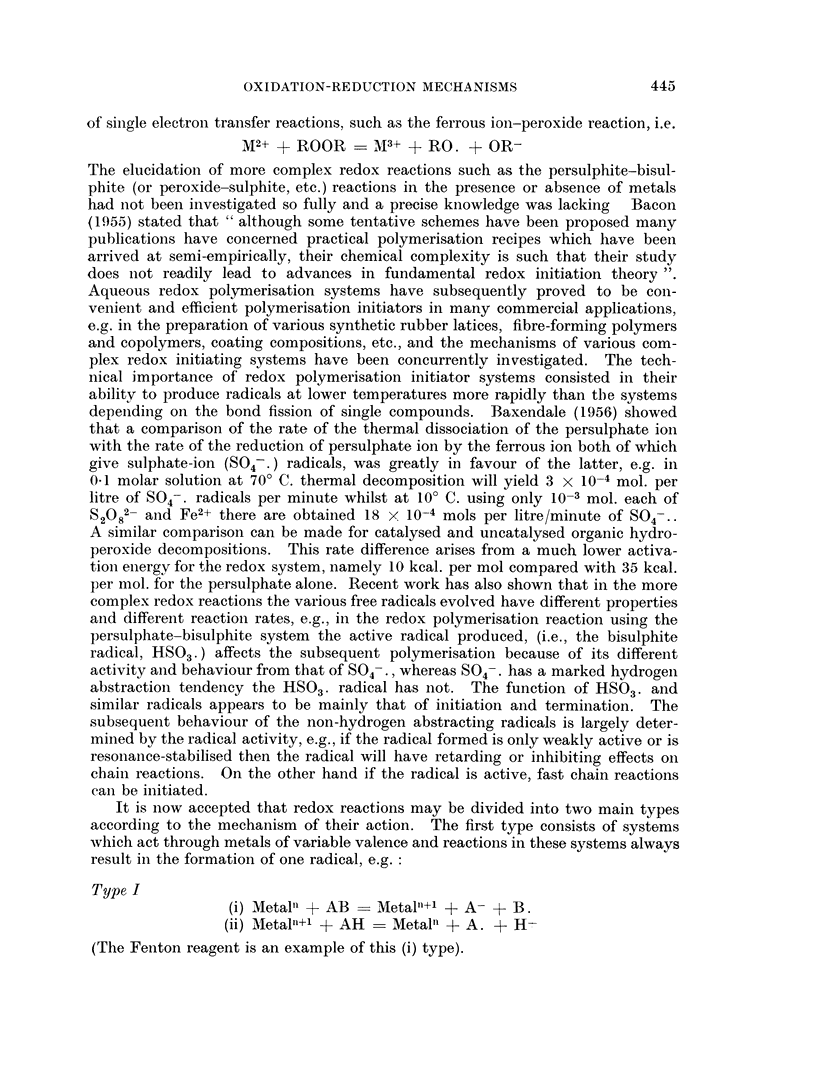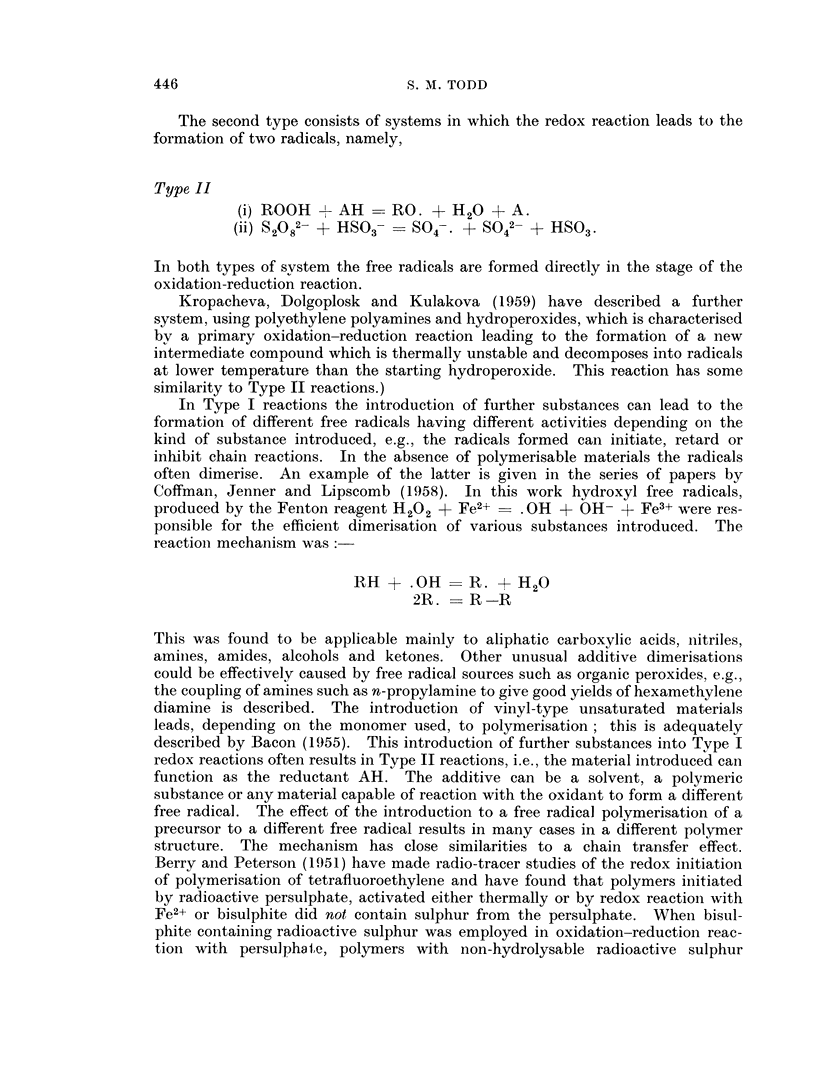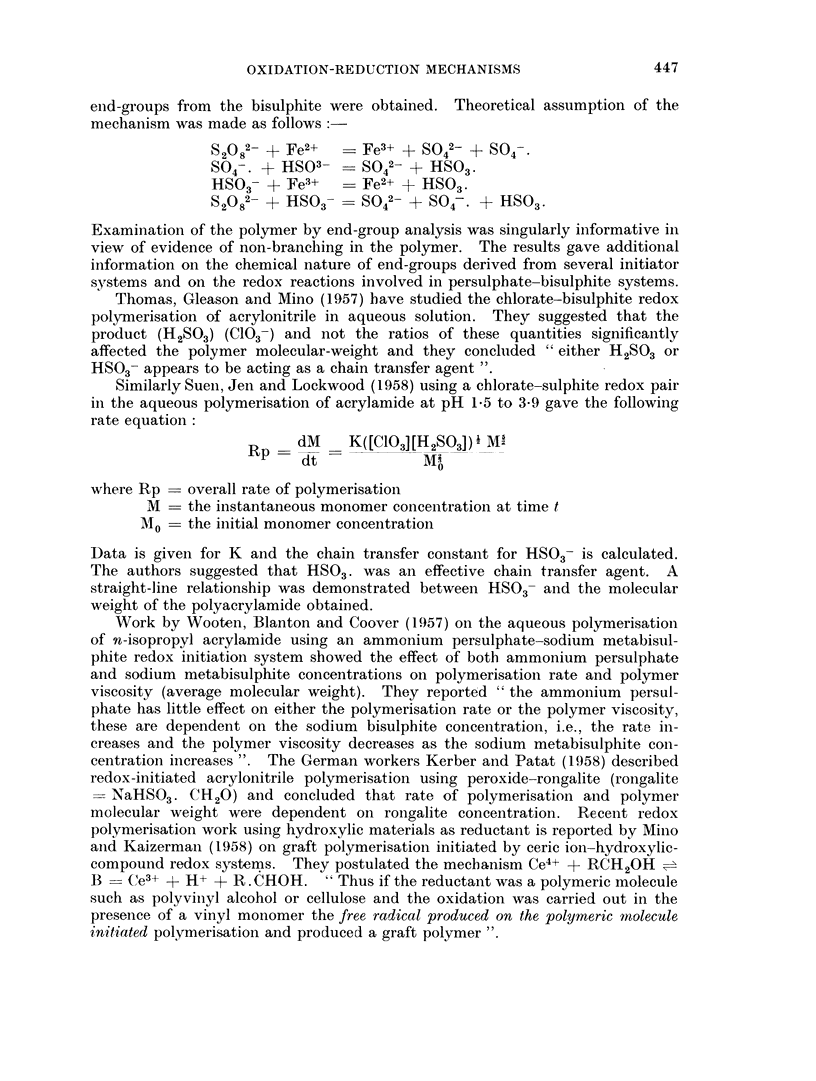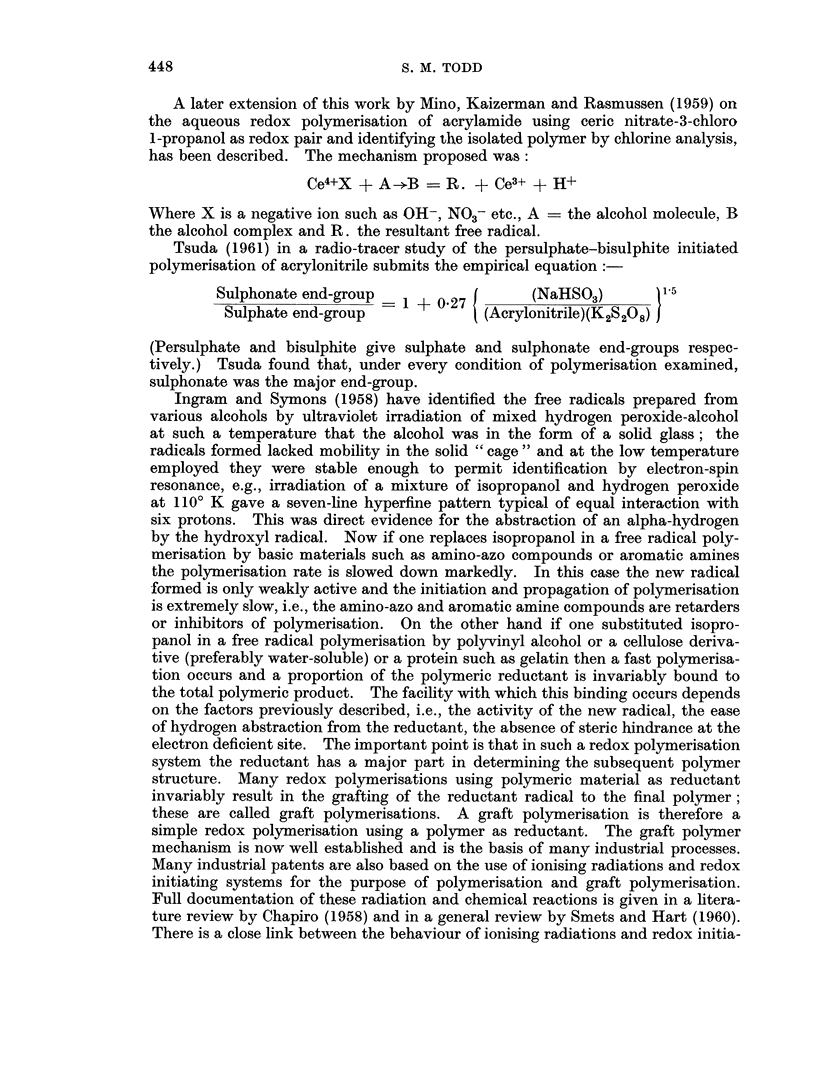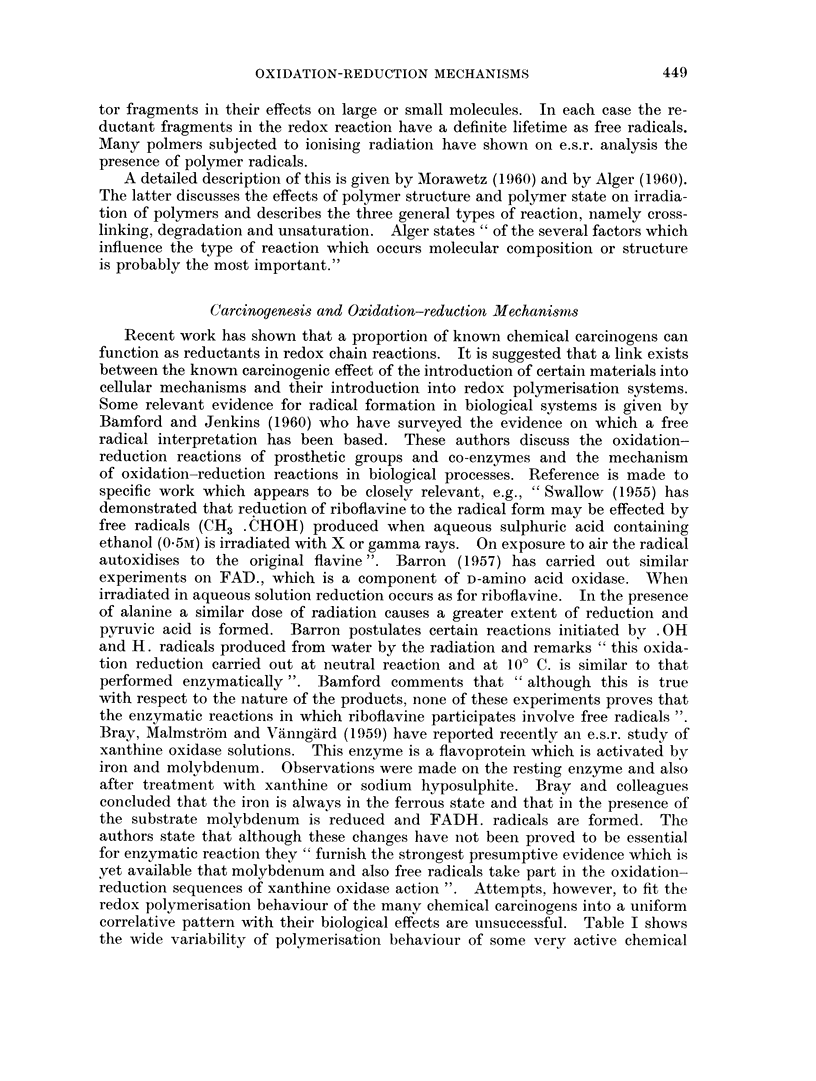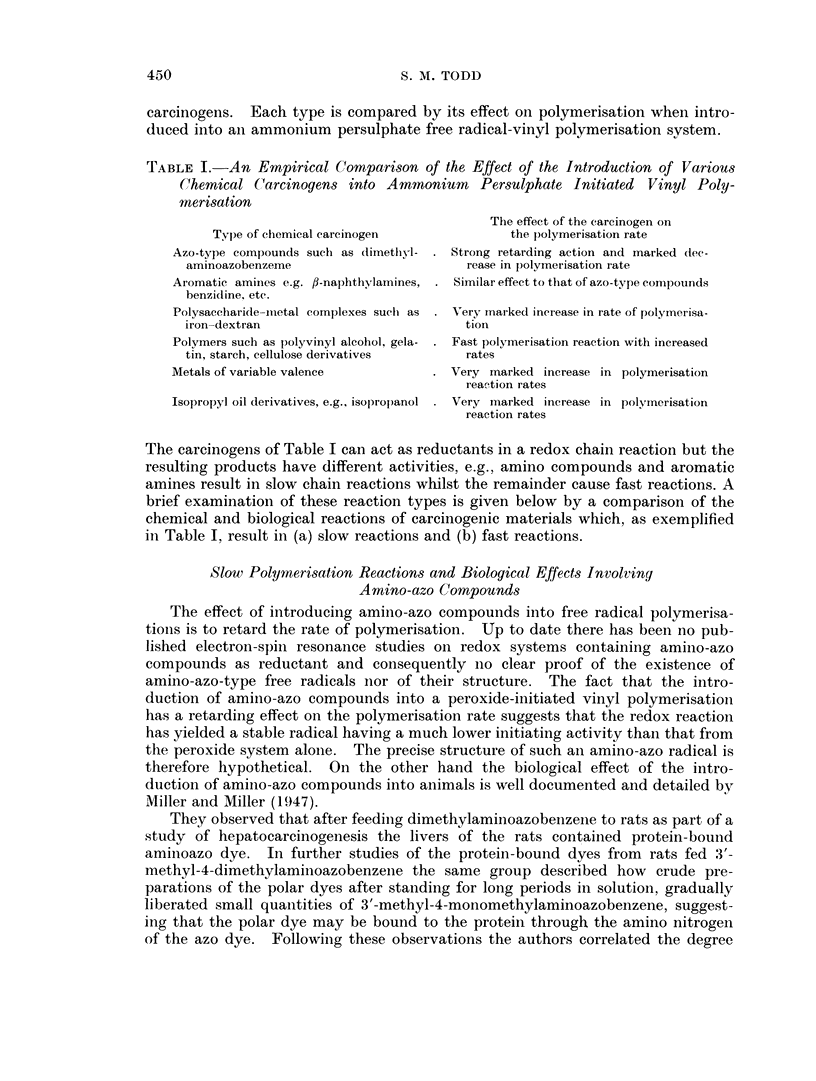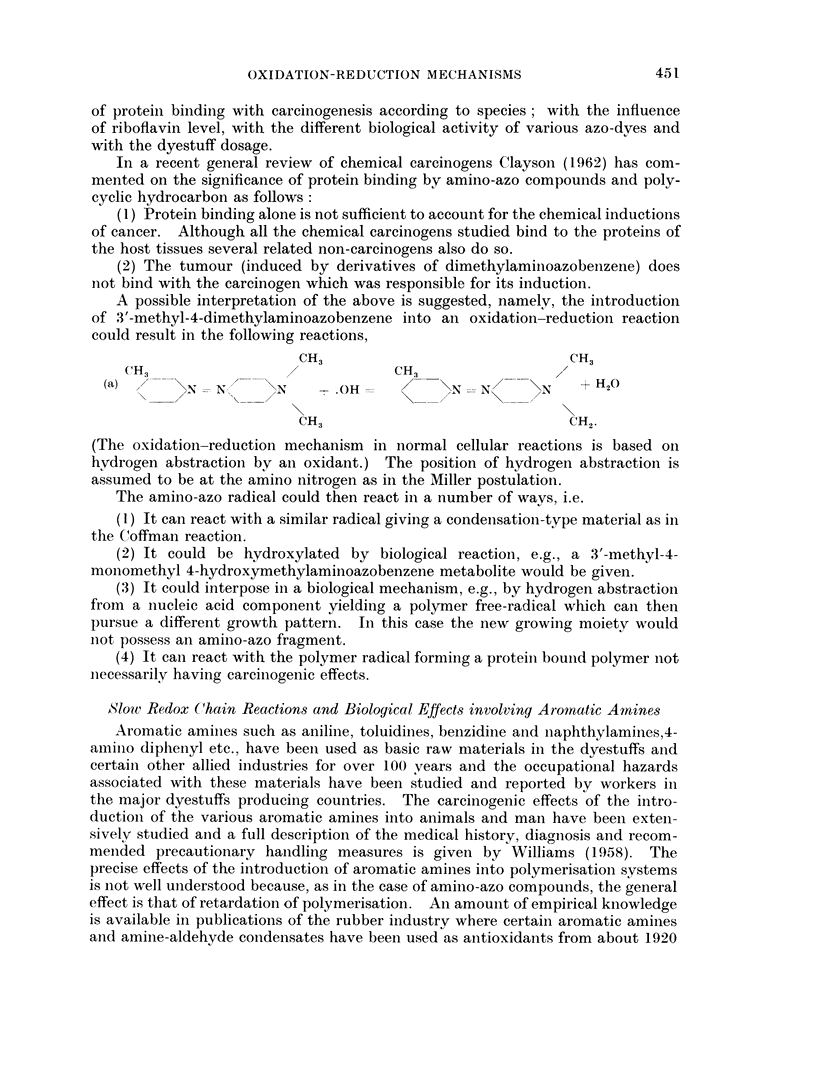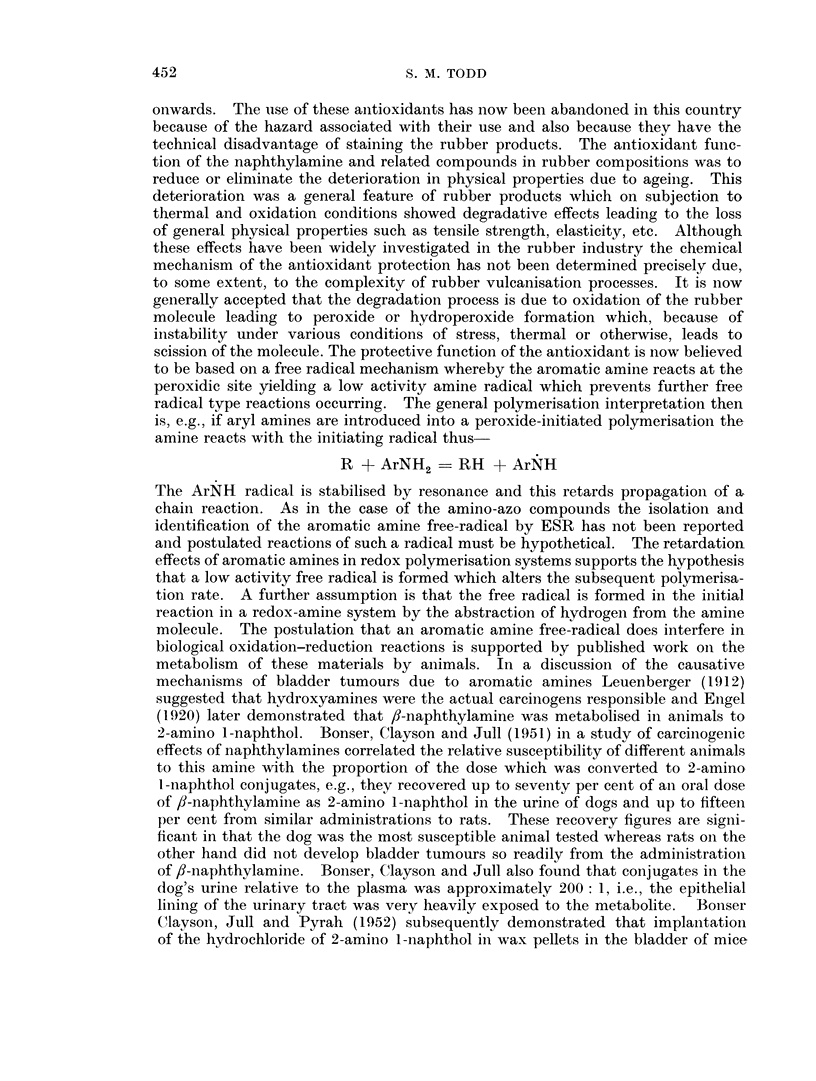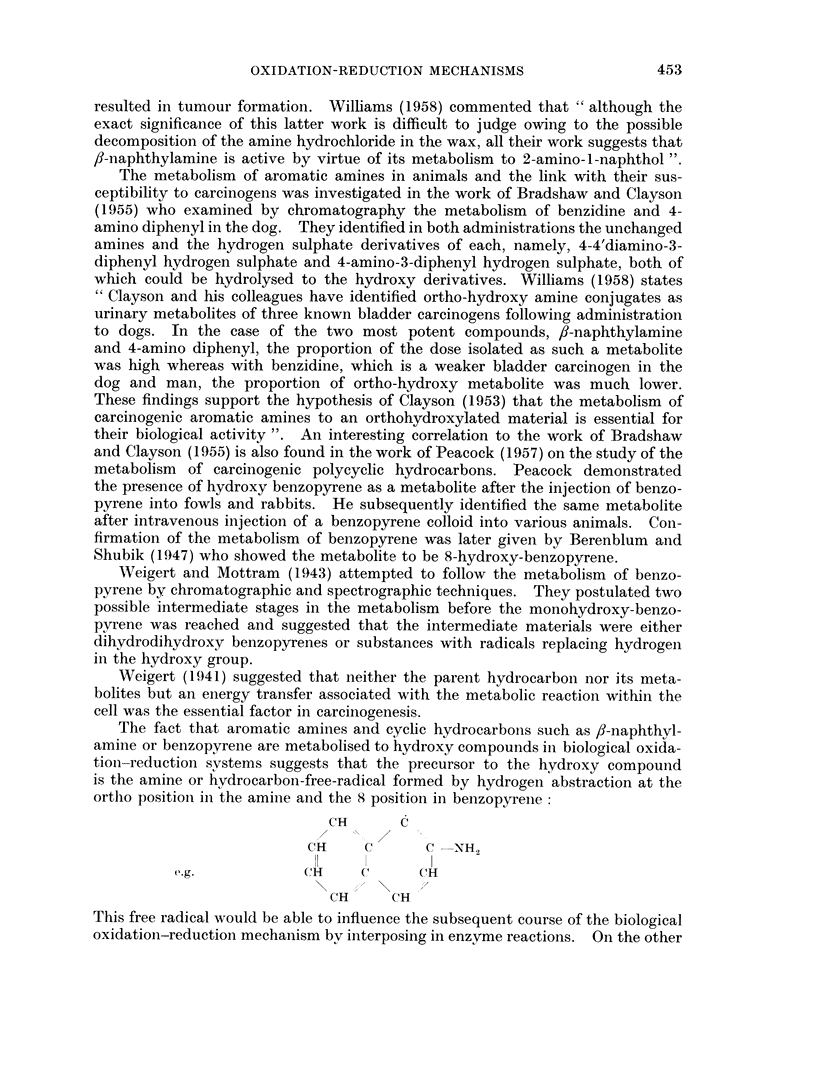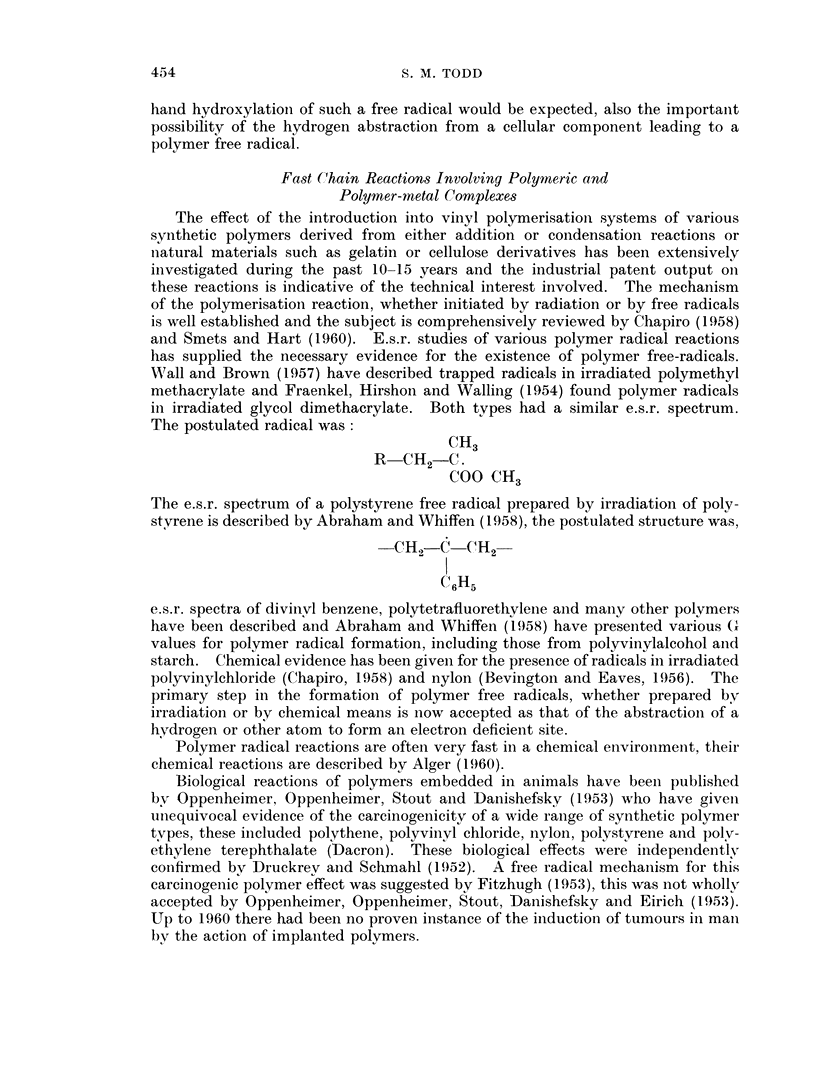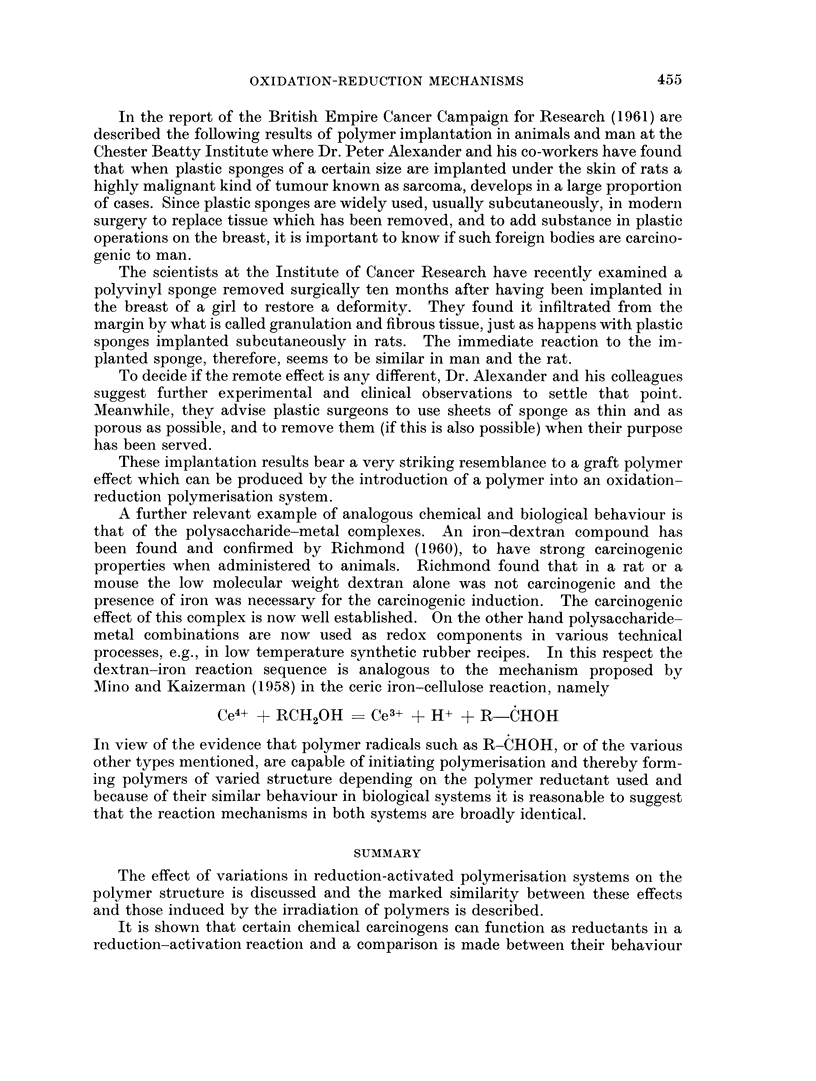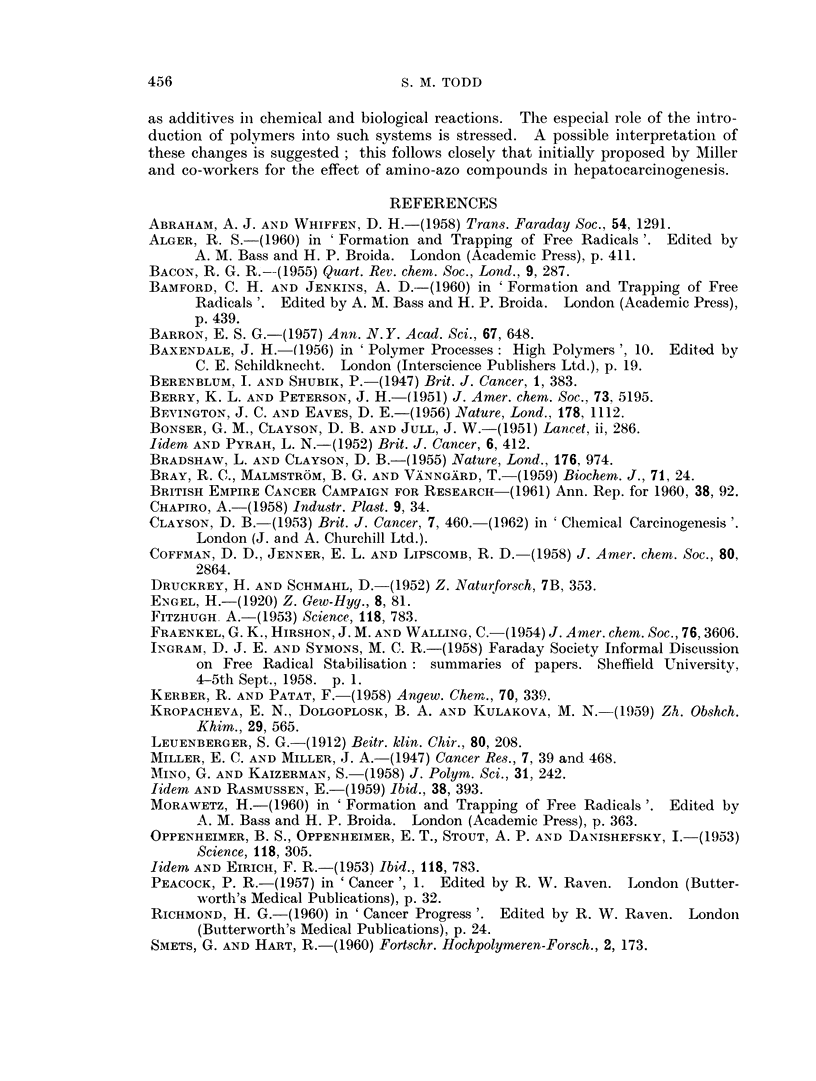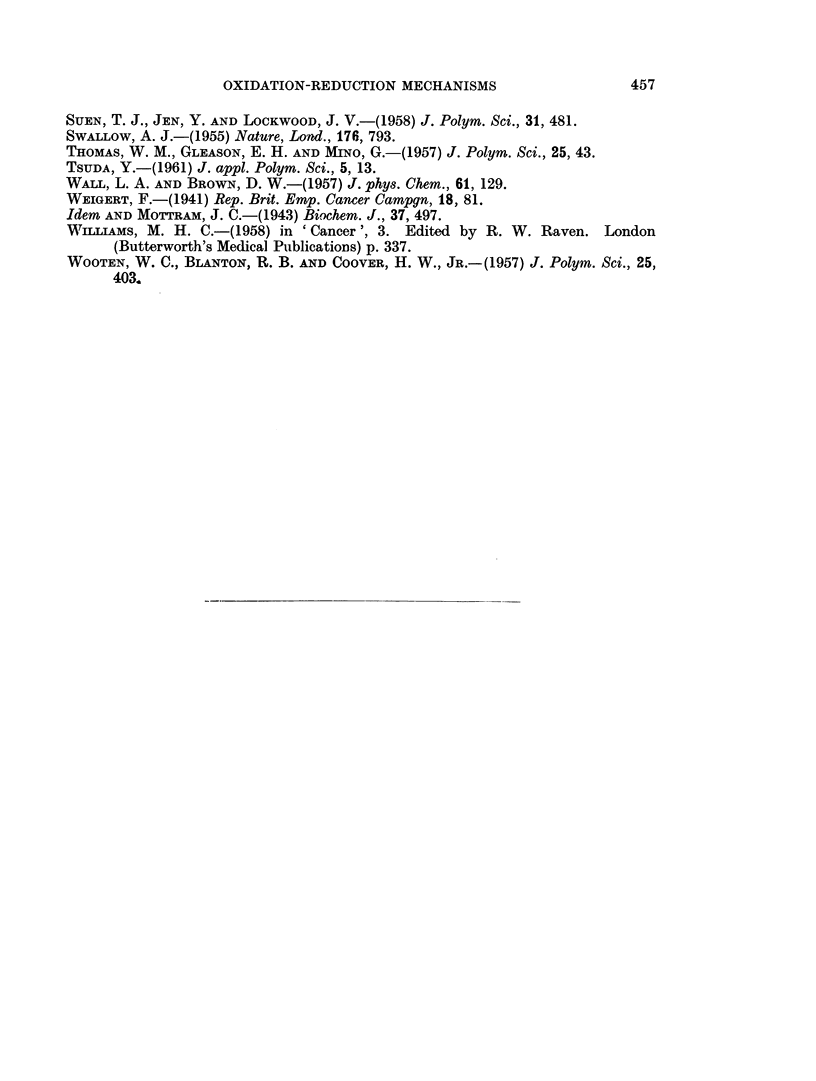# Some Speculations on Oxidation-Reduction Mechanisms and their Significance in the Induction of Cancer

**DOI:** 10.1038/bjc.1965.53

**Published:** 1965-06

**Authors:** S. M. Todd


### SOME SPECULATIONS ON OXIDATION–REDUCTION MECHANISMS AND THEIR SIGNIFICANCE IN THE INDUCTION OF CANCER

S. M. TODD

*From the Department of Chemistry, The University, Manchester*

Received for publication August 31, 1964

RECENT work on the more complex oxidation–reduction reactions has allowed the early interpretations of the reaction mechanisms to be extended. An integration of theory has occurred, partly due to contributions from the newer branches of polymerisation investigation and partly to the development of instrumental techniques. For example :

(i) The expansion of work on radiation chemistry has resulted in an extensive knowledge of the effects of radiation on many substances (including cellular materials and natural and synthetic polymers) and the structural changes caused by the irradiation have been widely investigated and reported. Marked correlation between radiation effects and free radical mechanisms has been observed.

(ii) The use of analytical techniques such as electron spin resonance (e.s.r.) and radio-tracer studies of polymerisation reactions has given a clearer understanding of polymerisation behaviour. Various redox polymerisation systems have been investigated and their mechanisms determined. This has led to an identification of the precise role of oxidant and reductant in various redox chain reactions.

The function of the reductant in redox polymerisations, whether it be a large (polymeric) or small molecule has been found to be important because of its dominant effect on the rate of polymerisation and the structure of the polymeric product. Substances introduced into a redox polymerisation reaction, which were formerly regarded solely as chain transfer agents, can function as reductants in the initial redox reaction yielding free radicals which can initiate polymerisation. The facility with which this occurs mainly depends on the structure and activity of the new radical and the activation energy requirement for the subsequent initiation and propagation reactions. It is suggested that many chemical carcinogens can function as reductants in redox chain reactions. A marked similarity exists between the chemical redox reactions and their effects in polymerisation mechanisms and the effects of the introduction of certain chemical carcinogens into biological systems.

### *The Influence of Oxidation–reduction Initiation Mechanisms on Polymerisation Rate and Polymer Structure*

The initial discovery of the oxidation–reduction (redox) effect in polymerisation systems and subsequent related work is described by Bacon (1955), who reviews published work on redox reactions up to the early 1950's and shows that the initial interpretation of these in this country was mainly established on the results of single electron transfer reactions, such as the ferrous ion–peroxide reaction, i.e.

$$M^{2+} + ROOR = M^{3+} + RO. + OR^-$$

The elucidation of more complex redox reactions such as the persulphite–bisulphite (or peroxide–sulphite, etc.) reactions in the presence or absence of metals had not been investigated so fully and a precise knowledge was lacking Bacon (1955) stated that " although some tentative schemes have been proposed many publications have concerned practical polymerisation recipes which have been arrived at semi-empirically, their chemical complexity is such that their study does not readily lead to advances in fundamental redox initiation theory ". Aqueous redox polymerisation systems have subsequently proved to be convenient and efficient polymerisation initiators in many commercial applications, e.g. in the preparation of various synthetic rubber latices, fibre-forming polymers and copolymers, coating compositions, etc., and the mechanisms of various complex redox initiating systems have been concurrently investigated. The technical importance of redox polymerisation initiator systems consisted in their ability to produce radicals at lower temperatures more rapidly than the systems depending on the bond fission of single compounds. Baxendale (1956) showed that a comparison of the rate of the thermal dissociation of the persulphate ion with the rate of the reduction of persulphate ion by the ferrous ion both of which give sulphate-ion ($SO_4^-$.) radicals, was greatly in favour of the latter, e.g. in 0·1 molar solution at 70° C. thermal decomposition will yield $3 \times 10^{-4}$ mol. per litre of $SO_4^-$. radicals per minute whilst at 10° C. using only $10^{-3}$ mol. each of $S_2O_8^{2-}$ and $Fe^{2+}$ there are obtained $18 \times 10^{-4}$ mols per litre/minute of $SO_4^-$.. A similar comparison can be made for catalysed and uncatalysed organic hydroperoxide decompositions. This rate difference arises from a much lower activation energy for the redox system, namely 10 kcal. per mol compared with 35 kcal. per mol. for the persulphate alone. Recent work has also shown that in the more complex redox reactions the various free radicals evolved have different properties and different reaction rates, e.g., in the redox polymerisation reaction using the persulphate–bisulphite system the active radical produced, (i.e., the bisulphite radical, $HSO_3$.) affects the subsequent polymerisation because of its different activity and behaviour from that of $SO_4^-$., whereas $SO_4^-$. has a marked hydrogen abstraction tendency the $HSO_3$. radical has not. The function of $HSO_3$. and similar radicals appears to be mainly that of initiation and termination. The subsequent behaviour of the non-hydrogen abstracting radicals is largely determined by the radical activity, e.g., if the radical formed is only weakly active or is resonance-stabilised then the radical will have retarding or inhibiting effects on chain reactions. On the other hand if the radical is active, fast chain reactions can be initiated.

It is now accepted that redox reactions may be divided into two main types according to the mechanism of their action. The first type consists of systems which act through metals of variable valence and reactions in these systems always result in the formation of one radical, e.g. :

*Type I*

$$\text{(i) } Metal^{n} + AB = Metal^{n+1} + A^- + B.$$

$$\text{(ii) } Metal^{n+1} + AH = Metal^{n} + A. + H^+$$

(The Fenton reagent is an example of this (i) type).

The second type consists of systems in which the redox reaction leads to the formation of two radicals, namely,

*Type II*

$$\text{(i) } ROOH + AH = RO. + H_2O + A.$$

$$\text{(ii) } S_2O_8^{2-} + HSO_3^- = SO_4^-. + SO_4^{2-} + HSO_3.$$

In both types of system the free radicals are formed directly in the stage of the oxidation-reduction reaction.

Kropacheva, Dolgoplosk and Kulakova (1959) have described a further system, using polyethylene polyamines and hydroperoxides, which is characterised by a primary oxidation–reduction reaction leading to the formation of a new intermediate compound which is thermally unstable and decomposes into radicals at lower temperature than the starting hydroperoxide. This reaction has some similarity to Type II reactions.)

In Type I reactions the introduction of further substances can lead to the formation of different free radicals having different activities depending on the kind of substance introduced, e.g., the radicals formed can initiate, retard or inhibit chain reactions. In the absence of polymerisable materials the radicals often dimerise. An example of the latter is given in the series of papers by Coffman, Jenner and Lipscomb (1958). In this work hydroxyl free radicals, produced by the Fenton reagent $H_2O_2 + Fe^{2+} = .OH + OH^- + Fe^{3+}$ were responsible for the efficient dimerisation of various substances introduced. The reaction mechanism was :—

$$RH + .OH = R. + H_2O$$
$$2R. = R{-}R$$

This was found to be applicable mainly to aliphatic carboxylic acids, nitriles, amines, amides, alcohols and ketones. Other unusual additive dimerisations could be effectively caused by free radical sources such as organic peroxides, e.g., the coupling of amines such as *n*-propylamine to give good yields of hexamethylene diamine is described. The introduction of vinyl-type unsaturated materials leads, depending on the monomer used, to polymerisation ; this is adequately described by Bacon (1955). This introduction of further substances into Type I redox reactions often results in Type II reactions, i.e., the material introduced can function as the reductant AH. The additive can be a solvent, a polymeric substance or any material capable of reaction with the oxidant to form a different free radical. The effect of the introduction to a free radical polymerisation of a precursor to a different free radical results in many cases in a different polymer structure. The mechanism has close similarities to a chain transfer effect. Berry and Peterson (1951) have made radio-tracer studies of the redox initiation of polymerisation of tetrafluoroethylene and have found that polymers initiated by radioactive persulphate, activated either thermally or by redox reaction with $Fe^{2+}$ or bisulphite did *not* contain sulphur from the persulphate. When bisulphite containing radioactive sulphur was employed in oxidation–reduction reaction with persulphate, polymers with non-hydrolysable radioactive sulphur end-groups from the bisulphite were obtained. Theoretical assumption of the mechanism was made as follows :—

$$S_2O_8^{2-} + Fe^{2+} = Fe^{3+} + SO_4^{2-} + SO_4^{-}.$$

$$SO_4^{-}. + HSO^{3-} = SO_4^{2-} + HSO_3.$$

$$HSO_3^{-} + Fe^{3+} = Fe^{2+} + HSO_3.$$

$$S_2O_8^{2-} + HSO_3^{-} = SO_4^{2-} + SO_4^{-}. + HSO_3.$$

Examination of the polymer by end-group analysis was singularly informative in view of evidence of non-branching in the polymer. The results gave additional information on the chemical nature of end-groups derived from several initiator systems and on the redox reactions involved in persulphate–bisulphite systems.

Thomas, Gleason and Mino (1957) have studied the chlorate–bisulphite redox polymerisation of acrylonitrile in aqueous solution. They suggested that the product ($H_2SO_3$) ($ClO_3^-$) and not the ratios of these quantities significantly affected the polymer molecular-weight and they concluded " either $H_2SO_3$ or $HSO_3^-$ appears to be acting as a chain transfer agent ".

Similarly Suen, Jen and Lockwood (1958) using a chlorate–sulphite redox pair in the aqueous polymerisation of acrylamide at pH 1·5 to 3·9 gave the following rate equation :

$$Rp = \frac{dM}{dt} = \frac{K([ClO_3][H_2SO_3])^{\frac{1}{2}} M^{\frac{5}{2}}}{M_0^{\frac{2}{3}}}$$

where Rp = overall rate of polymerisation
M = the instantaneous monomer concentration at time $t$
$M_0$ = the initial monomer concentration

Data is given for K and the chain transfer constant for $HSO_3^-$ is calculated. The authors suggested that $HSO_3$. was an effective chain transfer agent. A straight-line relationship was demonstrated between $HSO_3^-$ and the molecular weight of the polyacrylamide obtained.

Work by Wooten, Blanton and Coover (1957) on the aqueous polymerisation of *n*-isopropyl acrylamide using an ammonium persulphate–sodium metabisulphite redox initiation system showed the effect of both ammonium persulphate and sodium metabisulphite concentrations on polymerisation rate and polymer viscosity (average molecular weight). They reported " the ammonium persulphate has little effect on either the polymerisation rate or the polymer viscosity, these are dependent on the sodium bisulphite concentration, i.e., the rate increases and the polymer viscosity decreases as the sodium metabisulphite concentration increases ". The German workers Kerber and Patat (1958) described redox-initiated acrylonitrile polymerisation using peroxide–rongalite (rongalite = $NaHSO_3 . CH_2O$) and concluded that rate of polymerisation and polymer molecular weight were dependent on rongalite concentration. Recent redox polymerisation work using hydroxylic materials as reductant is reported by Mino and Kaizerman (1958) on graft polymerisation initiated by ceric ion–hydroxylic-compound redox systems. They postulated the mechanism $Ce^{4+} + RCH_2OH \rightleftharpoons B = Ce^{3+} + H^+ + R.\dot{C}HOH$. " Thus if the reductant was a polymeric molecule such as polyvinyl alcohol or cellulose and the oxidation was carried out in the presence of a vinyl monomer the *free radical produced on the polymeric molecule initiated* polymerisation and produced a graft polymer ".

A later extension of this work by Mino, Kaizerman and Rasmussen (1959) on the aqueous redox polymerisation of acrylamide using ceric nitrate-3-chloro 1-propanol as redox pair and identifying the isolated polymer by chlorine analysis, has been described. The mechanism proposed was :

$$Ce^{4+}X + A \rightarrow B = R. + Ce^{3+} + H^{+}$$

Where X is a negative ion such as $OH^-$, $NO_3^-$ etc., A = the alcohol molecule, B the alcohol complex and R. the resultant free radical.

Tsuda (1961) in a radio-tracer study of the persulphate–bisulphite initiated polymerisation of acrylonitrile submits the empirical equation :—

$$\frac{\text{Sulphonate end-group}}{\text{Sulphate end-group}} = 1 + 0{\cdot}27 \left\{ \frac{(NaHSO_3)}{(\text{Acrylonitrile})(K_2S_2O_8)} \right\}^{1{\cdot}5}$$

(Persulphate and bisulphite give sulphate and sulphonate end-groups respectively.) Tsuda found that, under every condition of polymerisation examined, sulphonate was the major end-group.

Ingram and Symons (1958) have identified the free radicals prepared from various alcohols by ultraviolet irradiation of mixed hydrogen peroxide-alcohol at such a temperature that the alcohol was in the form of a solid glass ; the radicals formed lacked mobility in the solid " cage " and at the low temperature employed they were stable enough to permit identification by electron-spin resonance, e.g., irradiation of a mixture of isopropanol and hydrogen peroxide at 110° K gave a seven-line hyperfine pattern typical of equal interaction with six protons. This was direct evidence for the abstraction of an alpha-hydrogen by the hydroxyl radical. Now if one replaces isopropanol in a free radical polymerisation by basic materials such as amino-azo compounds or aromatic amines the polymerisation rate is slowed down markedly. In this case the new radical formed is only weakly active and the initiation and propagation of polymerisation is extremely slow, i.e., the amino-azo and aromatic amine compounds are retarders or inhibitors of polymerisation. On the other hand if one substituted isopropanol in a free radical polymerisation by polyvinyl alcohol or a cellulose derivative (preferably water-soluble) or a protein such as gelatin then a fast polymerisation occurs and a proportion of the polymeric reductant is invariably bound to the total polymeric product. The facility with which this binding occurs depends on the factors previously described, i.e., the activity of the new radical, the ease of hydrogen abstraction from the reductant, the absence of steric hindrance at the electron deficient site. The important point is that in such a redox polymerisation system the reductant has a major part in determining the subsequent polymer structure. Many redox polymerisations using polymeric material as reductant invariably result in the grafting of the reductant radical to the final polymer ; these are called graft polymerisations. A graft polymerisation is therefore a simple redox polymerisation using a polymer as reductant. The graft polymer mechanism is now well established and is the basis of many industrial processes. Many industrial patents are also based on the use of ionising radiations and redox initiating systems for the purpose of polymerisation and graft polymerisation. Full documentation of these radiation and chemical reactions is given in a literature review by Chapiro (1958) and in a general review by Smets and Hart (1960). There is a close link between the behaviour of ionising radiations and redox initiator fragments in their effects on large or small molecules. In each case the reductant fragments in the redox reaction have a definite lifetime as free radicals. Many polmers subjected to ionising radiation have shown on e.s.r. analysis the presence of polymer radicals.

A detailed description of this is given by Morawetz (1960) and by Alger (1960). The latter discusses the effects of polymer structure and polymer state on irradiation of polymers and describes the three general types of reaction, namely cross-linking, degradation and unsaturation. Alger states " of the several factors which influence the type of reaction which occurs molecular composition or structure is probably the most important."

### *Carcinogenesis and Oxidation–reduction Mechanisms*

Recent work has shown that a proportion of known chemical carcinogens can function as reductants in redox chain reactions. It is suggested that a link exists between the known carcinogenic effect of the introduction of certain materials into cellular mechanisms and their introduction into redox polymerisation systems. Some relevant evidence for radical formation in biological systems is given by Bamford and Jenkins (1960) who have surveyed the evidence on which a free radical interpretation has been based. These authors discuss the oxidation–reduction reactions of prosthetic groups and co-enzymes and the mechanism of oxidation–reduction reactions in biological processes. Reference is made to specific work which appears to be closely relevant, e.g., " Swallow (1955) has demonstrated that reduction of riboflavine to the radical form may be effected by free radicals ($CH_3$ .$\dot{C}$HOH) produced when aqueous sulphuric acid containing ethanol (0·5M) is irradiated with X or gamma rays. On exposure to air the radical autoxidises to the original flavine ". Barron (1957) has carried out similar experiments on FAD., which is a component of D-amino acid oxidase. When irradiated in aqueous solution reduction occurs as for riboflavine. In the presence of alanine a similar dose of radiation causes a greater extent of reduction and pyruvic acid is formed. Barron postulates certain reactions initiated by .OH and H. radicals produced from water by the radiation and remarks " this oxidation reduction carried out at neutral reaction and at 10° C. is similar to that performed enzymatically ". Bamford comments that " although this is true with respect to the nature of the products, none of these experiments proves that the enzymatic reactions in which riboflavine participates involve free radicals ". Bray, Malmström and Vänngård (1959) have reported recently an e.s.r. study of xanthine oxidase solutions. This enzyme is a flavoprotein which is activated by iron and molybdenum. Observations were made on the resting enzyme and also after treatment with xanthine or sodium hyposulphite. Bray and colleagues concluded that the iron is always in the ferrous state and that in the presence of the substrate molybdenum is reduced and FADH. radicals are formed. The authors state that although these changes have not been proved to be essential for enzymatic reaction they " furnish the strongest presumptive evidence which is yet available that molybdenum and also free radicals take part in the oxidation–reduction sequences of xanthine oxidase action ". Attempts, however, to fit the redox polymerisation behaviour of the many chemical carcinogens into a uniform correlative pattern with their biological effects are unsuccessful. Table I shows the wide variability of polymerisation behaviour of some very active chemical carcinogens. Each type is compared by its effect on polymerisation when introduced into an ammonium persulphate free radical-vinyl polymerisation system.

TABLE I.—*An Empirical Comparison of the Effect of the Introduction of Various Chemical Carcinogens into Ammonium Persulphate Initiated Vinyl Polymerisation*

| Type of chemical carcinogen | The effect of the carcinogen on the polymerisation rate |
|---|---|
| Azo-type compounds such as dimethylaminoazobenzeme | Strong retarding action and marked decrease in polymerisation rate |
| Aromatic amines e.g. $\beta$-naphthylamines, benzidine, etc. | Similar effect to that of azo-type compounds |
| Polysaccharide-metal complexes such as iron–dextran | Very marked increase in rate of polymerisation |
| Polymers such as polyvinyl alcohol, gelatin, starch, cellulose derivatives | Fast polymerisation reaction with increased rates |
| Metals of variable valence | Very marked increase in polymerisation reaction rates |
| Isopropyl oil derivatives, e.g., isopropanol | Very marked increase in polymerisation reaction rates |

The carcinogens of Table I can act as reductants in a redox chain reaction but the resulting products have different activities, e.g., amino compounds and aromatic amines result in slow chain reactions whilst the remainder cause fast reactions. A brief examination of these reaction types is given below by a comparison of the chemical and biological reactions of carcinogenic materials which, as exemplified in Table I, result in (a) slow reactions and (b) fast reactions.

### *Slow Polymerisation Reactions and Biological Effects Involving Amino-azo Compounds*

The effect of introducing amino-azo compounds into free radical polymerisations is to retard the rate of polymerisation. Up to date there has been no published electron-spin resonance studies on redox systems containing amino-azo compounds as reductant and consequently no clear proof of the existence of amino-azo-type free radicals nor of their structure. The fact that the introduction of amino-azo compounds into a peroxide-initiated vinyl polymerisation has a retarding effect on the polymerisation rate suggests that the redox reaction has yielded a stable radical having a much lower initiating activity than that from the peroxide system alone. The precise structure of such an amino-azo radical is therefore hypothetical. On the other hand the biological effect of the introduction of amino-azo compounds into animals is well documented and detailed by Miller and Miller (1947).

They observed that after feeding dimethylaminoazobenzene to rats as part of a study of hepatocarcinogenesis the livers of the rats contained protein-bound aminoazo dye. In further studies of the protein-bound dyes from rats fed 3′-methyl-4-dimethylaminoazobenzene the same group described how crude preparations of the polar dyes after standing for long periods in solution, gradually liberated small quantities of 3′-methyl-4-monomethylaminoazobenzene, suggesting that the polar dye may be bound to the protein through the amino nitrogen of the azo dye. Following these observations the authors correlated the degree of protein binding with carcinogenesis according to species ; with the influence of riboflavin level, with the different biological activity of various azo-dyes and with the dyestuff dosage.

In a recent general review of chemical carcinogens Clayson (1962) has commented on the significance of protein binding by amino-azo compounds and polycyclic hydrocarbon as follows :

(1) Protein binding alone is not sufficient to account for the chemical inductions of cancer. Although all the chemical carcinogens studied bind to the proteins of the host tissues several related non-carcinogens also do so.

(2) The tumour (induced by derivatives of dimethylaminoazobenzene) does not bind with the carcinogen which was responsible for its induction.

A possible interpretation of the above is suggested, namely, the introduction of 3′-methyl-4-dimethylaminoazobenzene into an oxidation–reduction reaction could result in the following reactions,

(a) 

(The oxidation–reduction mechanism in normal cellular reactions is based on hydrogen abstraction by an oxidant.) The position of hydrogen abstraction is assumed to be at the amino nitrogen as in the Miller postulation.

The amino-azo radical could then react in a number of ways, i.e.

(1) It can react with a similar radical giving a condensation-type material as in the Coffman reaction.

(2) It could be hydroxylated by biological reaction, e.g., a 3′-methyl-4-monomethyl 4-hydroxymethylaminoazobenzene metabolite would be given.

(3) It could interpose in a biological mechanism, e.g., by hydrogen abstraction from a nucleic acid component yielding a polymer free-radical which can then pursue a different growth pattern. In this case the new growing moiety would not possess an amino-azo fragment.

(4) It can react with the polymer radical forming a protein bound polymer not necessarily having carcinogenic effects.

*Slow Redox Chain Reactions and Biological Effects involving Aromatic Amines*

Aromatic amines such as aniline, toluidines, benzidine and naphthylamines,4-amino diphenyl etc., have been used as basic raw materials in the dyestuffs and certain other allied industries for over 100 years and the occupational hazards associated with these materials have been studied and reported by workers in the major dyestuffs producing countries. The carcinogenic effects of the introduction of the various aromatic amines into animals and man have been extensively studied and a full description of the medical history, diagnosis and recommended precautionary handling measures is given by Williams (1958). The precise effects of the introduction of aromatic amines into polymerisation systems is not well understood because, as in the case of amino-azo compounds, the general effect is that of retardation of polymerisation. An amount of empirical knowledge is available in publications of the rubber industry where certain aromatic amines and amine-aldehyde condensates have been used as antioxidants from about 1920

onwards. The use of these antioxidants has now been abandoned in this country because of the hazard associated with their use and also because they have the technical disadvantage of staining the rubber products. The antioxidant function of the naphthylamine and related compounds in rubber compositions was to reduce or eliminate the deterioration in physical properties due to ageing. This deterioration was a general feature of rubber products which on subjection to thermal and oxidation conditions showed degradative effects leading to the loss of general physical properties such as tensile strength, elasticity, etc. Although these effects have been widely investigated in the rubber industry the chemical mechanism of the antioxidant protection has not been determined precisely due, to some extent, to the complexity of rubber vulcanisation processes. It is now generally accepted that the degradation process is due to oxidation of the rubber molecule leading to peroxide or hydroperoxide formation which, because of instability under various conditions of stress, thermal or otherwise, leads to scission of the molecule. The protective function of the antioxidant is now believed to be based on a free radical mechanism whereby the aromatic amine reacts at the peroxidic site yielding a low activity amine radical which prevents further free radical type reactions occurring. The general polymerisation interpretation then is, e.g., if aryl amines are introduced into a peroxide-initiated polymerisation the amine reacts with the initiating radical thus—

$$\mathrm{R} + \mathrm{ArNH_2} = \mathrm{RH} + \mathrm{Ar\dot{N}H}$$

The $\mathrm{Ar\dot{N}H}$ radical is stabilised by resonance and this retards propagation of a chain reaction. As in the case of the amino-azo compounds the isolation and identification of the aromatic amine free-radical by ESR has not been reported and postulated reactions of such a radical must be hypothetical. The retardation effects of aromatic amines in redox polymerisation systems supports the hypothesis that a low activity free radical is formed which alters the subsequent polymerisation rate. A further assumption is that the free radical is formed in the initial reaction in a redox-amine system by the abstraction of hydrogen from the amine molecule. The postulation that an aromatic amine free-radical does interfere in biological oxidation–reduction reactions is supported by published work on the metabolism of these materials by animals. In a discussion of the causative mechanisms of bladder tumours due to aromatic amines Leuenberger (1912) suggested that hydroxyamines were the actual carcinogens responsible and Engel (1920) later demonstrated that $\beta$-naphthylamine was metabolised in animals to 2-amino 1-naphthol. Bonser, Clayson and Jull (1951) in a study of carcinogenic effects of naphthylamines correlated the relative susceptibility of different animals to this amine with the proportion of the dose which was converted to 2-amino 1-naphthol conjugates, e.g., they recovered up to seventy per cent of an oral dose of $\beta$-naphthylamine as 2-amino 1-naphthol in the urine of dogs and up to fifteen per cent from similar administrations to rats. These recovery figures are significant in that the dog was the most susceptible animal tested whereas rats on the other hand did not develop bladder tumours so readily from the administration of $\beta$-naphthylamine. Bonser, Clayson and Jull also found that conjugates in the dog's urine relative to the plasma was approximately 200 : 1, i.e., the epithelial lining of the urinary tract was very heavily exposed to the metabolite. Bonser Clayson, Jull and Pyrah (1952) subsequently demonstrated that implantation of the hydrochloride of 2-amino 1-naphthol in wax pellets in the bladder of mice resulted in tumour formation. Williams (1958) commented that " although the exact significance of this latter work is difficult to judge owing to the possible decomposition of the amine hydrochloride in the wax, all their work suggests that $\beta$-naphthylamine is active by virtue of its metabolism to 2-amino-1-naphthol ".

The metabolism of aromatic amines in animals and the link with their susceptibility to carcinogens was investigated in the work of Bradshaw and Clayson (1955) who examined by chromatography the metabolism of benzidine and 4-amino diphenyl in the dog. They identified in both administrations the unchanged amines and the hydrogen sulphate derivatives of each, namely, 4-4'diamino-3-diphenyl hydrogen sulphate and 4-amino-3-diphenyl hydrogen sulphate, both of which could be hydrolysed to the hydroxy derivatives. Williams (1958) states " Clayson and his colleagues have identified ortho-hydroxy amine conjugates as urinary metabolites of three known bladder carcinogens following administration to dogs. In the case of the two most potent compounds, $\beta$-naphthylamine and 4-amino diphenyl, the proportion of the dose isolated as such a metabolite was high whereas with benzidine, which is a weaker bladder carcinogen in the dog and man, the proportion of ortho-hydroxy metabolite was much lower. These findings support the hypothesis of Clayson (1953) that the metabolism of carcinogenic aromatic amines to an orthohydroxylated material is essential for their biological activity ". An interesting correlation to the work of Bradshaw and Clayson (1955) is also found in the work of Peacock (1957) on the study of the metabolism of carcinogenic polycyclic hydrocarbons. Peacock demonstrated the presence of hydroxy benzopyrene as a metabolite after the injection of benzopyrene into fowls and rabbits. He subsequently identified the same metabolite after intravenous injection of a benzopyrene colloid into various animals. Confirmation of the metabolism of benzopyrene was later given by Berenblum and Shubik (1947) who showed the metabolite to be 8-hydroxy-benzopyrene.

Weigert and Mottram (1943) attempted to follow the metabolism of benzopyrene by chromatographic and spectrographic techniques. They postulated two possible intermediate stages in the metabolism before the monohydroxy-benzopyrene was reached and suggested that the intermediate materials were either dihydrodihydroxy benzopyrenes or substances with radicals replacing hydrogen in the hydroxy group.

Weigert (1941) suggested that neither the parent hydrocarbon nor its metabolites but an energy transfer associated with the metabolic reaction within the cell was the essential factor in carcinogenesis.

The fact that aromatic amines and cyclic hydrocarbons such as $\beta$-naphthylamine or benzopyrene are metabolised to hydroxy compounds in biological oxidation–reduction systems suggests that the precursor to the hydroxy compound is the amine or hydrocarbon-free-radical formed by hydrogen abstraction at the ortho position in the amine and the 8 position in benzopyrene :

e.g.

This free radical would be able to influence the subsequent course of the biological oxidation–reduction mechanism by interposing in enzyme reactions. On the other hand hydroxylation of such a free radical would be expected, also the important possibility of the hydrogen abstraction from a cellular component leading to a polymer free radical.

*Fast Chain Reactions Involving Polymeric and Polymer-metal Complexes*

The effect of the introduction into vinyl polymerisation systems of various synthetic polymers derived from either addition or condensation reactions or natural materials such as gelatin or cellulose derivatives has been extensively investigated during the past 10–15 years and the industrial patent output on these reactions is indicative of the technical interest involved. The mechanism of the polymerisation reaction, whether initiated by radiation or by free radicals is well established and the subject is comprehensively reviewed by Chapiro (1958) and Smets and Hart (1960). E.s.r. studies of various polymer radical reactions has supplied the necessary evidence for the existence of polymer free-radicals. Wall and Brown (1957) have described trapped radicals in irradiated polymethyl methacrylate and Fraenkel, Hirshon and Walling (1954) found polymer radicals in irradiated glycol dimethacrylate. Both types had a similar e.s.r. spectrum. The postulated radical was :

$$R\text{—}CH_2\text{—}\overset{\displaystyle CH_3}{\underset{\displaystyle COO\ CH_3}{C}}.$$

The e.s.r. spectrum of a polystyrene free radical prepared by irradiation of polystyrene is described by Abraham and Whiffen (1958), the postulated structure was,

$$\text{—}CH_2\text{—}\underset{\displaystyle C_6H_5}{\underset{|}{\dot{C}}}\text{—}CH_2\text{—}$$

e.s.r. spectra of divinyl benzene, polytetrafluorethylene and many other polymers have been described and Abraham and Whiffen (1958) have presented various G values for polymer radical formation, including those from polyvinylalcohol and starch. Chemical evidence has been given for the presence of radicals in irradiated polyvinylchloride (Chapiro, 1958) and nylon (Bevington and Eaves, 1956). The primary step in the formation of polymer free radicals, whether prepared by irradiation or by chemical means is now accepted as that of the abstraction of a hydrogen or other atom to form an electron deficient site.

Polymer radical reactions are often very fast in a chemical environment, their chemical reactions are described by Alger (1960).

Biological reactions of polymers embedded in animals have been published by Oppenheimer, Oppenheimer, Stout and Danishefsky (1953) who have given unequivocal evidence of the carcinogenicity of a wide range of synthetic polymer types, these included polythene, polyvinyl chloride, nylon, polystyrene and polyethylene terephthalate (Dacron). These biological effects were independently confirmed by Druckrey and Schmahl (1952). A free radical mechanism for this carcinogenic polymer effect was suggested by Fitzhugh (1953), this was not wholly accepted by Oppenheimer, Oppenheimer, Stout, Danishefsky and Eirich (1953). Up to 1960 there had been no proven instance of the induction of tumours in man by the action of implanted polymers.

In the report of the British Empire Cancer Campaign for Research (1961) are described the following results of polymer implantation in animals and man at the Chester Beatty Institute where Dr. Peter Alexander and his co-workers have found that when plastic sponges of a certain size are implanted under the skin of rats a highly malignant kind of tumour known as sarcoma, develops in a large proportion of cases. Since plastic sponges are widely used, usually subcutaneously, in modern surgery to replace tissue which has been removed, and to add substance in plastic operations on the breast, it is important to know if such foreign bodies are carcinogenic to man.

The scientists at the Institute of Cancer Research have recently examined a polyvinyl sponge removed surgically ten months after having been implanted in the breast of a girl to restore a deformity. They found it infiltrated from the margin by what is called granulation and fibrous tissue, just as happens with plastic sponges implanted subcutaneously in rats. The immediate reaction to the implanted sponge, therefore, seems to be similar in man and the rat.

To decide if the remote effect is any different, Dr. Alexander and his colleagues suggest further experimental and clinical observations to settle that point. Meanwhile, they advise plastic surgeons to use sheets of sponge as thin and as porous as possible, and to remove them (if this is also possible) when their purpose has been served.

These implantation results bear a very striking resemblance to a graft polymer effect which can be produced by the introduction of a polymer into an oxidation–reduction polymerisation system.

A further relevant example of analogous chemical and biological behaviour is that of the polysaccharide–metal complexes. An iron–dextran compound has been found and confirmed by Richmond (1960), to have strong carcinogenic properties when administered to animals. Richmond found that in a rat or a mouse the low molecular weight dextran alone was not carcinogenic and the presence of iron was necessary for the carcinogenic induction. The carcinogenic effect of this complex is now well established. On the other hand polysaccharide–metal combinations are now used as redox components in various technical processes, e.g., in low temperature synthetic rubber recipes. In this respect the dextran–iron reaction sequence is analogous to the mechanism proposed by Mino and Kaizerman (1958) in the ceric iron–cellulose reaction, namely

$$Ce^{4+} + RCH_2OH = Ce^{3+} + H^+ + R\text{—}\dot{C}HOH$$

In view of the evidence that polymer radicals such as $R\text{–}\dot{C}HOH$, or of the various other types mentioned, are capable of initiating polymerisation and thereby forming polymers of varied structure depending on the polymer reductant used and because of their similar behaviour in biological systems it is reasonable to suggest that the reaction mechanisms in both systems are broadly identical.

### SUMMARY

The effect of variations in reduction-activated polymerisation systems on the polymer structure is discussed and the marked similarity between these effects and those induced by the irradiation of polymers is described.

It is shown that certain chemical carcinogens can function as reductants in a reduction–activation reaction and a comparison is made between their behaviour as additives in chemical and biological reactions. The especial role of the introduction of polymers into such systems is stressed. A possible interpretation of these changes is suggested ; this follows closely that initially proposed by Miller and co-workers for the effect of amino-azo compounds in hepatocarcinogenesis.

### REFERENCES

Abraham, A. J. and Whiffen, D. H.—(1958) *Trans. Faraday Soc.*, **54**, 1291.

Alger, R. S.—(1960) in ‘ Formation and Trapping of Free Radicals ’. Edited by A. M. Bass and H. P. Broida. London (Academic Press), p. 411.

Bacon, R. G. R.—(1955) *Quart. Rev. chem. Soc., Lond.*, **9**, 287.

Bamford, C. H. and Jenkins, A. D.—(1960) in ‘ Formation and Trapping of Free Radicals ’. Edited by A. M. Bass and H. P. Broida. London (Academic Press), p. 439.

Barron, E. S. G.—(1957) *Ann. N.Y. Acad. Sci.*, **67**, 648.

Baxendale, J. H.—(1956) in ‘ Polymer Processes : High Polymers ’, 10. Edited by C. E. Schildknecht. London (Interscience Publishers Ltd.), p. 19.

Berenblum, I. and Shubik, P.—(1947) *Brit. J. Cancer*, **1**, 383.

Berry, K. L. and Peterson, J. H.—(1951) *J. Amer. chem. Soc.*, **73**, 5195.

Bevington, J. C. and Eaves, D. E.—(1956) *Nature, Lond.*, **178**, 1112.

Bonser, G. M., Clayson, D. B. and Jull, J. W.—(1951) *Lancet*, ii, 286.

*Iidem* and Pyrah, L. N.—(1952) *Brit. J. Cancer*, **6**, 412.

Bradshaw, L. and Clayson, D. B.—(1955) *Nature, Lond.*, **176**, 974.

Bray, R. C., Malmström, B. G. and Vänngård, T.—(1959) *Biochem. J.*, **71**, 24.

British Empire Cancer Campaign for Research—(1961) Ann. Rep. for 1960, **38**, 92.

Chapiro, A.—(1958) *Industr. Plast.* **9**, 34.

Clayson, D. B.—(1953) *Brit. J. Cancer*, **7**, 460.—(1962) in ‘ Chemical Carcinogenesis ’. London (J. and A. Churchill Ltd.).

Coffman, D. D., Jenner, E. L. and Lipscomb, R. D.—(1958) *J. Amer. chem. Soc.*, **80**, 2864.

Druckrey, H. and Schmahl, D.—(1952) *Z. Naturforsch*, **7**B, 353.

Engel, H.—(1920) *Z. Gew-Hyg.*, **8**, 81.

Fitzhugh. A.—(1953) *Science*, **118**, 783.

Fraenkel, G. K., Hirshon, J. M. and Walling, C.—(1954) *J. Amer. chem. Soc.*, **76**, 3606.

Ingram, D. J. E. and Symons, M. C. R.—(1958) Faraday Society Informal Discussion on Free Radical Stabilisation : summaries of papers. Sheffield University, 4–5th Sept., 1958. p. 1.

Kerber, R. and Patat, F.—(1958) *Angew. Chem.*, **70**, 339.

Kropacheva, E. N., Dolgoplosk, B. A. and Kulakova, M. N.—(1959) *Zh. Obshch. Khim.*, **29**, 565.

Leuenberger, S. G.—(1912) *Beitr. klin. Chir.*, **80**, 208.

Miller, E. C. and Miller, J. A.—(1947) *Cancer Res.*, **7**, 39 and 468.

Mino, G. and Kaizerman, S.—(1958) *J. Polym. Sci.*, **31**, 242.

*Iidem* and Rasmussen, E.—(1959) *Ibid.*, **38**, 393.

Morawetz, H.—(1960) in ‘ Formation and Trapping of Free Radicals ’. Edited by A. M. Bass and H. P. Broida. London (Academic Press), p. 363.

Oppenheimer, B. S., Oppenheimer, E. T., Stout, A. P. and Danishefsky, I.—(1953) *Science*, **118**, 305.

*Iidem* and Eirich, F. R.—(1953) *Ibid.*, **118**, 783.

Peacock, P. R.—(1957) in ‘ Cancer ’, 1. Edited by R. W. Raven. London (Butterworth's Medical Publications), p. 32.

Richmond, H. G.—(1960) in ‘ Cancer Progress ’. Edited by R. W. Raven. London (Butterworth's Medical Publications), p. 24.

Smets, G. and Hart, R.—(1960) *Fortschr. Hochpolymeren-Forsch.*, **2**, 173.

SUEN, T. J., JEN, Y. AND LOCKWOOD, J. V.—(1958) *J. Polym. Sci.*, **31**, 481.
SWALLOW, A. J.—(1955) *Nature, Lond.*, **176**, 793.
THOMAS, W. M., GLEASON, E. H. AND MINO, G.—(1957) *J. Polym. Sci.*, **25**, 43.
TSUDA, Y.—(1961) *J. appl. Polym. Sci.*, **5**, 13.
WALL, L. A. AND BROWN, D. W.—(1957) *J. phys. Chem.*, **61**, 129.
WEIGERT, F.—(1941) *Rep. Brit. Emp. Cancer Campgn*, **18**, 81.
*Idem* AND MOTTRAM, J. C.—(1943) *Biochem. J.*, **37**, 497.
WILLIAMS, M. H. C.—(1958) in ‘Cancer’, 3. Edited by R. W. Raven. London (Butterworth's Medical Publications) p. 337.
WOOTEN, W. C., BLANTON, R. B. AND COOVER, H. W., JR.—(1957) *J. Polym. Sci.*, **25**, 403.